# Supplementary material for: Ultrahigh-Q guided mode resonances in an All-dielectric metasurface
Source: Nat Commun. 2023 Jun 10;14:3433. doi: 10.1038/s41467-023-39227-5 (PMC10257673; doi:10.1038/s41467-023-39227-5)
Supplement: Supplementary file 1 — Supplementary Information [file 41467_2023_39227_MOESM1_ESM.pdf]

Supplementary Materials for Ultrahigh-Q Guided Mode Resonances in an All-Dielectric Metasurface

Lujun Huang[1#*], Rong Jin[2,3,4#], Chaobiao Zhou[5#], Guanhai Li[2,3,4*], Lei Xu[6], Adam Overvig[7], Fu Deng[1], Xiaoshuang Chen[2,3,4], Wei Lu[2,3,4], Andrea Alu[7,8*], and Andrey E Miroshnichenko[1*]

[1] School of Engineering and Information Technology, University of New South Wales, Canberra, Northcott Drive, ACT, 2600, Australia

[2]State Key Laboratory of Infrared Physics, Shanghai Institute of Technical Physics, Chinese Academy of Sciences, 500 Yu Tian Road, Shanghai, 200083, China

[3]Hangzhou Institute for Advanced Study, University of Chinese Academy of Sciences, No.1 SubLane Xiangshan, Hangzhou, 310024, China

[4]Shanghai Research Center for Quantum Sciences, 99 Xiupu Road, Shanghai, 201315, China

[5]School of Physics and Mechatronic Engineering, Guizhou Minzu University, Guiyang, 550025, China

[6]Advanced Optics and Photonics Laboratory, Department of Engineering, School of Science Technology, Nottingham Trent University, Nottingham NG11 8NS, UK

[7]Photonics Initiative, Advanced Science Research Center, City University of New York, New York, NY 10031, United States of America

[8]Physics Program, Graduate Center, City University of New York, New York, NY 10016, United States of America

# These authors contributed equally to this work

**ljhuang@mail.sitp.ac.cn, ghli0120@mail.sitp.ac.cn, aalu@gc.cuny.edu, andrey.miroshnichenko@unsw.edu.au*

Section 1-Fabry-Perot leaky modes in a three-layer structure

We consider a three-layer structure shown in Fig.1a. The thickness of the middle layer is L. Such a structure could support a number of Fabry-Perot leaky modes. Detailed derivations of their eigenvalues are given below. Note exp($-i\omega t$) is assumed during the following derivation process

Assume E_x—H_y and interface located at z=-L/2 and z=L/2

Solving the Maxwell equations, one can have the electric field as follows

$$E_x(z) = A\exp(+in_1k_0z),\quad z>L/2 \tag{1a}$$

$$E_x(z) = B.\exp(-in_2k_0z)+C.\exp(+in_2k_0z),\ -L/2<z<L/2 \tag{1b}$$

$$E_x(z) = D\exp(-in_3k_0z),\quad z<-L/2 \tag{1c}$$

Also, the magnetic field H can be obtained by following equation

$$i\omega\mu_0 H_y = \frac{\partial E_x}{\partial z} \tag{2}$$

Substituting eq.(1) into eq.(2), we will have

$$H_y(z) = A.(n_1\mu_0/c).\exp(+in_1k_0z),\ z>L/2 \tag{3a}$$

$$H_y(z) = B.(-n_2\mu_0/c).\exp(-in_2k_0z) + C.(n_2\mu_0/c).\exp(+in_2k_0z),\ -L/2<z<L/2 \tag{3b}$$

$$H_y(z) = D.(-n_3\mu_0/c).\exp(-in_3k_0z),\ z>L/2 \tag{3c}$$

By matching the continuity of boundary conditions at z=-L/2 and z=L/2,

$$A\exp(in_1k_0L/2) = B.\exp(-in_2k_0L/2) + C.\exp(+in_2k_0L/2) \quad (4a)$$
$$D\exp(in_3k_0L/2) = B.\exp(+in2k_0L/2) + C.\exp(-in_2k_0L/2) \quad (4b)$$
$$A.(n_1).\exp(in_1k_0L/2) = B.(-n_2).\exp(-in_2k_0L/2) + C.n_2.\exp(+in_2k_0L/2) \quad (4c)$$
$$D.(-n_3).\exp(in_3k_0L/2) = B.(-n_2).\exp(+in_2k_0L/2) + C.n_2.\exp(-in_2k_0L/2) \quad (4d)$$

$$(4a)*n_1-(4c): (n_1+n_2).\exp(-in_2k_0L/2).B + (n_1-n_2).\exp(+in_2k_0L/2).C = 0 \quad (5a)$$
$$(4b)*n_3+(4c): (n_3-n_2).\exp(+in_2k_0L/2).B + (n_3+n_2).\exp(-in_2k_0L/2).C = 0 \quad (5b)$$

Or

$$(n_1+n_2).\exp(-in_2k_0L/2).B = (n_2-n_1).\exp(+in_2k_0L/2).C \quad (6a)$$
$$(n_2-n_3).\exp(+in_2k_0L/2).B = (n_3+n_2).\exp(-in_2k_0L/2).C \quad (6b)$$

(6a)/6(b) gives us

$$(n_1+n_2)(n_2+n_3)/(n_2-n_1)/(n_2-n_3) = \exp(2in_2k_0L) \quad (7)$$

For example, for air/Si/SiO_2 with n_1=1, n_2=3.47 and n_3=1.46, the eigenvalues are

$$N = n_2k_0L = m\pi - 0.7452i (m=1,2,3\ldots) \quad (8)$$

Section 2-Band structure for three-layer waveguide structure and GMs

We again consider a three-layer waveguide structure as shown in Fig.S1a, where a high-index layer n_2 with thickness t is sandwiched between the super-substrate n_1 and substrate n_3. The waveguide dispersion for TE (E//y) and TM (H//y) have been derived in the textbook and are given as follows

$$\text{TE:}\ \tan(\beta_2 t) = \frac{\beta_2(\beta_1+\beta_3)}{\beta_2^2-\beta_1\beta_3} \quad (9)$$

$$\text{TM:}\ \tan(\beta_2 t) = \frac{n_2^2\beta_2(\beta_1+n_3^2\beta_3)}{n_3^2\beta_2^2-n_2^2\beta_1\beta_3} \quad (10)$$

Where $\beta_1 = \sqrt{k^2 - n_1^2k_0^2}$, $\beta_2 = \sqrt{n_2^2k_0^2 - k^2}$, $\beta_3 = \sqrt{k^2 - n_3^2k_0^2}$, and $k = \sqrt{k_x^2 + k_y^2}$.

Also, it is necessary to mention that there is a cut-off frequency for different guided modes.

$$\Omega_{cj} = \frac{c}{t\sqrt{n_2^2-n_3^2}}\left(\tan^{-1}\left[s\sqrt{\frac{n_3^2-n_1^2}{n_2^2-n_3^2}}\right] + j\pi\right)(j = 0,1,2\ldots) \quad (11)$$

Where $s = 1$ for TE and $s = n_2^2$, c is the speed of light in the vacuum.

To plot the band structure for such a waveguide system, we introduce an artificial but virtual periodic boundary condition. There are two ways of defining the periodic structure: 1D grating and 2D metasurface, as schematically shown in Fig.S2a-b. We first treat this waveguide system as a virtual grating with the period d. The band structure could be obtained by solving the transcendent equations (9) and (10). Fig.3 plots band structures of TE and TM modes for a real waveguide structure air/220 nm-Si/SiO_2. The refractive index of Si and SiO_2 are set as n_2=3.47 and n_3=1.46, respectively. We are particularly interested in the guided modes at Γ point because they can be treated as pseudo bound states in the continuum (GMs).

The GMs can be obtained by setting

$$k = \frac{2m\pi}{d} \tag{12}$$

Where $m = 1,2,3, ...$

Substituting Eq.(12) into Eqs. (9) and (10) gives us the eigenfrequencies of GMs

Generally speaking, GMs could be designed at any wavelength by choosing the artificial period. For example, the period is tuned to d=548 nm to support 1st GM at 1550 nm. Such a waveguide with d=548 nm also supports many other GMs for both TE and TM modes at different wavelengths (See Fig.3 and Fig.S4). Moreover, due to the square lattice, there are degeneracy for GMs labelled in Fig.S3. For example, for 1D TE case, GM-1 includes TE_{21} and TE_{31}, GM-2 includes TE_{22} and TE_{32}, GM-3 includes TE_{41} and TE_{51}. Their field profiles are shown in Fig.3a. For TM case, GM-4 include TM_{21} and TM_{31}, GM-5 includes TM_{22} and TM_{32}, GM-6 includes TE_{41} and TE_{51}. Their field profiles are presented in Fig.S4

For a 2D virtual metasurface with a square lattice, the degeneracy number is 4. The degeneracy could be lifted and reduced to 2 by applying different lattice constants dx and dy along the x- and y-axis, respectively. In fact, the virtual 1D grating can be treated as a special example of 2D metasurface by setting the lattice constant dy as infinity. Similarly, the GMs at Γ point can be obtained by setting

$$k = 2\pi\sqrt{\frac{m^2}{d_x^2} + \frac{n^2}{d_y^2}} \tag{13}$$

Where $m, n = 0,1,2,3, ...$

For square lattice with $d_x = d_y = d$, it becomes

$$k = \frac{2\pi}{d}\sqrt{m^2 + n^2} \tag{14}$$

Where $m, n = 0,1,2,3, ...$

Section 3-Perturbation theory for GMs

In this section, we analyze the effect of structural perturbation on the GMs. Again, we consider the three-layer waveguide structure shown in Fig.1a. For the sake of simplicity and without loss of generality, we treat this waveguide structure as a 1D virtual grating structure, its dielectric functions $\varepsilon_*(x, z)$ can be expressed as following forms

$$\varepsilon_*(x, z) = \varepsilon_*(x + d, z) = n_1^2 \ (z > t) \tag{15a}$$

$$\varepsilon_*(x, z) = \varepsilon_*(x + d, z) = n_2^2 \ (0 \le z \le t) \tag{15b}$$

$$\varepsilon_*(x, z) = \varepsilon_*(x + d, z) = n_3^2 \ (z < 0) \tag{15c}$$

For the TE polarization case (E//y), Ey, also denoted as u, should satisfy the Helmholtz equation

$$\frac{\partial^2 u}{\partial x^2} + \frac{\partial^2 u}{\partial y^2} + k_*^2 \varepsilon_* = 0 \tag{16}$$

Where $k_* = \omega_*/c$, ω is the eigenfrequency of the eigenmode and c is the speed of light.

Because we only consider the eigenmodes (also called as GMs in this work) at Γ point, its eigenfunction should satisfy

$$u(x,z) = u(x+d,z) \quad (17)$$

Here, we assume that $0 < k_* < 2\pi/d$.

Then, we introduce the perturbation layer on top of layer 2 to this system

$$\varepsilon(x,y) = \varepsilon_*(x,y) + \delta F(x,y) \quad (18)$$

Where δ is a small real parameter, $F(x,y)$ is the perturbation profiles, which is $F(x,y) = 1$ ($t < z < t + t_0, -\frac{w}{2} < x < w/2$) and $F(x,y) = 0$ for the else region.

After perturbation layer is added, GMs are converted into real leaky modes. Note that these leaky modes include symmetry protected BICs with anti-symmetric field profile and GMRs with symmetric field profiles. Here, we focus on studying the perturbation layer effect on the Q-factor of GMRs. We are interested in complex eigenfrequency ω and Q factor nearby GMs. They can be expanded as

$$u = u_* + u_1\delta + u_2\delta^2 + u_3\delta^3 + \cdots \quad (19a)$$

$$\omega = \omega_* + \omega_1\delta + \omega_2\delta^2 + \omega_3\delta^3 + \cdots \quad (19b)$$

$$k = k_* + k_1\delta + k_2\delta^2 + k_3\delta^3 + \cdots \quad (19c)$$

Where $k_j = \frac{\omega_j}{c}, j = 1,2,3$ ...

Substituting Eq.(17) into the Helmholtz equation and comparing the coefficients of $\delta^0, \delta^1, \delta^2$ give us

$$\mathcal{L}u_* = 0 \quad (20a)$$

$$\mathcal{L}u_1 = -(2k_*k_1\varepsilon_* + k_*^2F)u_* \quad (20b)$$

$$\mathcal{L}u_2 = -(2k_*k_1\varepsilon_* + k_*^2F)u_1 - (2k_*k_2\varepsilon_* + k_1^2\varepsilon_* + 2k_*k_1F)u_* \quad (20c)$$

Where $\mathcal{L} = \partial_x^2 + \partial_x^2 + k_*^2\varepsilon_*$

Due to the nature of leaky modes, u_j must satisfy the outgoing radiation condition when |z|→∞. Also, u_j is a periodic function. Note that Eq. 20b is inhomogeneous and singular. It is easy to prove that there is no solution u_1 unless the right side of Eq.20b is orthogonal to u_*. We define the whole region Ω as |x|<d/2, -∞<z<∞. We then multiply the complex conjugate of u_*on both sides and integrate on Ω, we have

$$\int \overline{u_*}\mathcal{L}u_1 \, dxdz = 0 \quad (21)$$

Then, we have

$$k_1 = \frac{\omega_1}{c} = -\frac{k_* \int F|u_*|^2 dxdz}{2\int \varepsilon_*|u_*|^2 dxdz} \quad (22)$$

Because F is real, k_1 is also real.

For $0 < k_* < 2\pi/d$, u_1 is mainly contributed by the zero-diffraction and contains only a single radiation channel. Thus, we have

$$u_1 \sim b_0^{\pm} e^{\pm ik_*x}, x \to \pm\infty \quad (23)$$

Where $b_0^{\pm}$ are nonzero coefficients.

We also derive the imaginary part of k_2 from the Eq. (18c),

$$Im(k_2) = \frac{Im(\omega_2)}{c} = -\frac{d(|b_0^+|^2 + |b_0^-|^2)}{2\int \varepsilon_*|u_*|^2 dxdz}\delta^2 \quad (24)$$

A detailed derivation of the above equation can be found in Ref. [3].

Then, the imaginary part of complex eigenfrequency becomes

$$Im(\omega_2) = -\frac{cd(|b_0^+|^2 + |b_0^-|^2)}{2\int \varepsilon_* |u_*|^2 dxdz}\delta^2 \tag{25}$$

And thus, we will have Q-factors

$$Q = \frac{k_* \int \varepsilon_* |u_*|^2 dxdz}{d(|b_0^+|^2 + |b_0^-|^2)}\delta^{-2} \tag{26}$$

Thus, the Q-factors of such GMRs are inversely proportional to the perturbation squared.

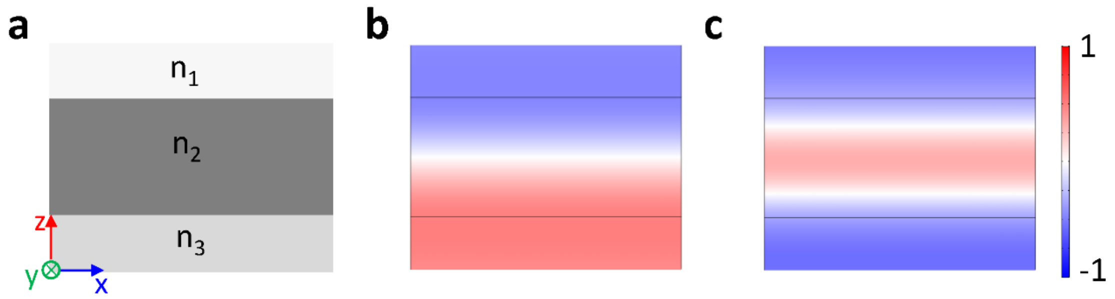

Figure S1. Fabry-Perot leaky modes in multilayer structure. a, schematic drawing of a three-layer thin film structure. b, Ey distribution of mode TEM12. c, Ey distribution of mode TEM13.

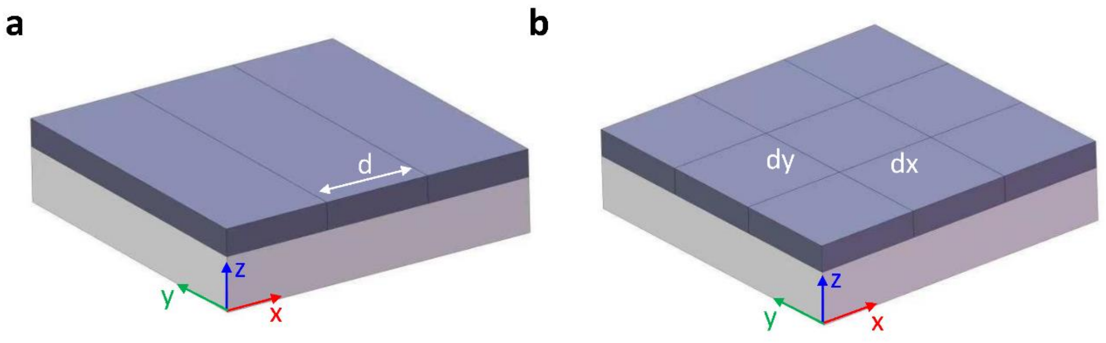

Figure S2. Virtual periodic structure for a three-layer waveguide system. a, virtual 1D grating structure with lattice constant d along the x-axis. b, virtual 2D metasurface with different lattice constants dx and dy along the x- and y-axis.

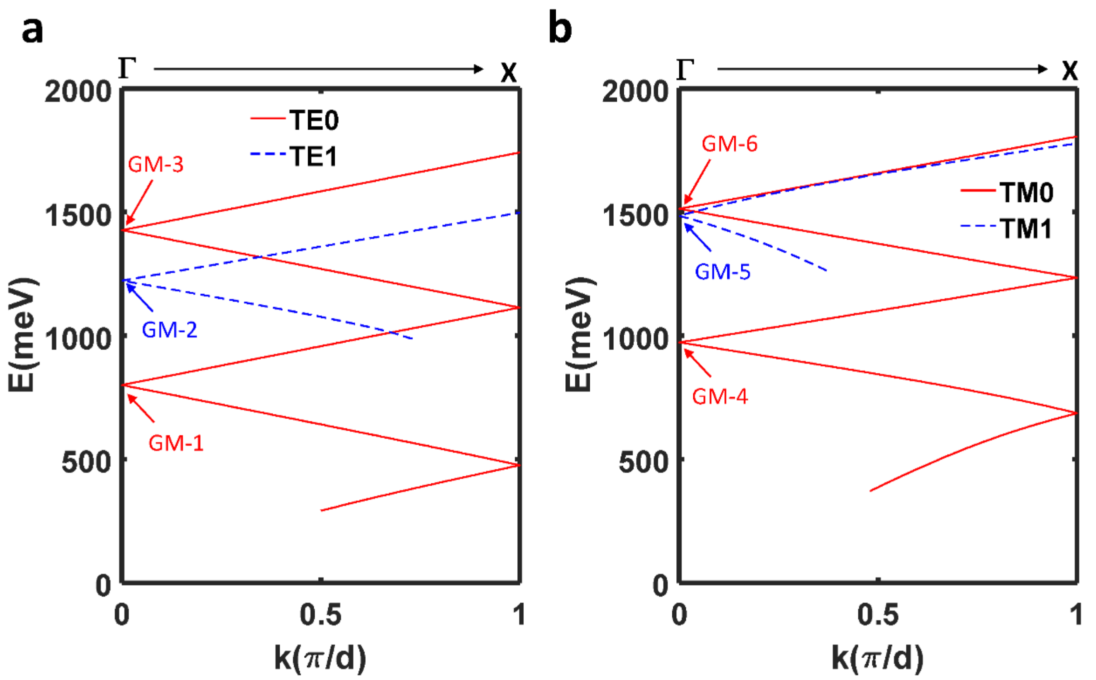

Figure S3. Dispersion relationship of TE and TM guided modes. a, Band structure of TE guided modes. b, Band structure of TM guided modes.

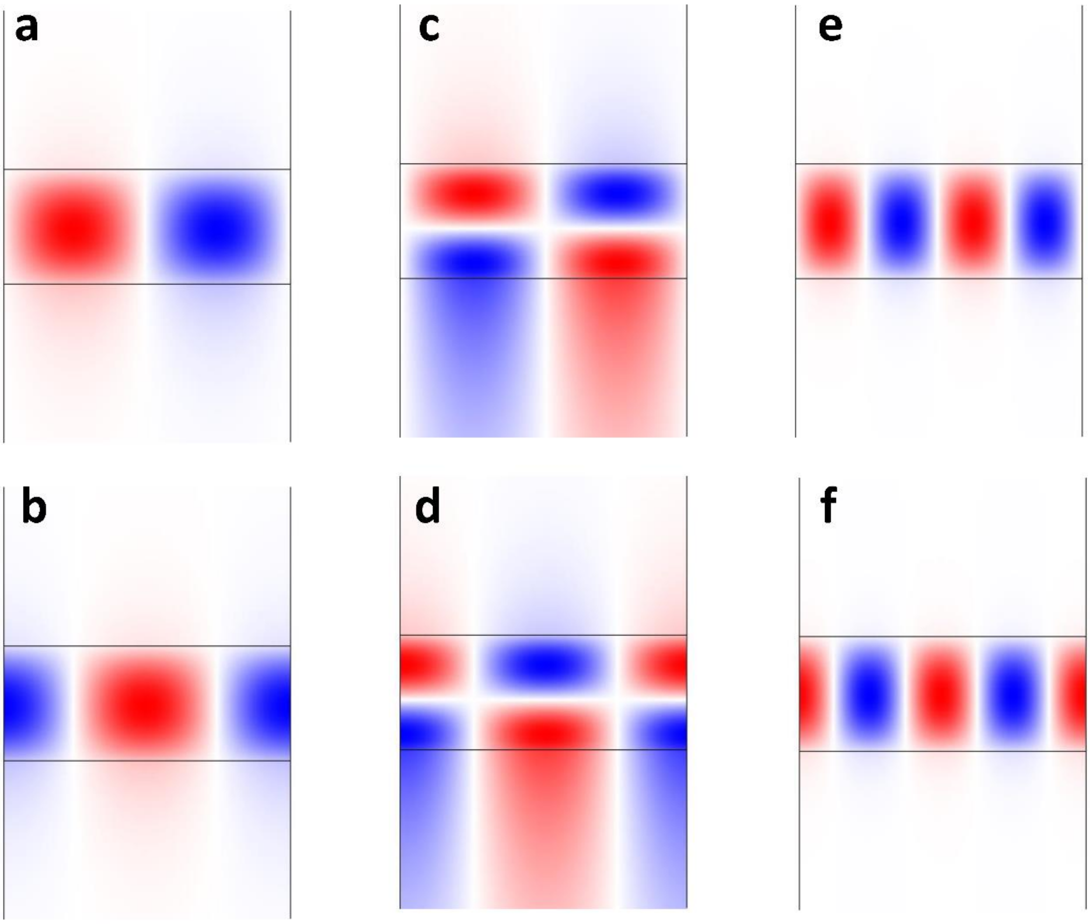

Figure S4. GMs for TM case. a, Hz distribution of GM TM21 mode. b, Hz distribution of GM TM31 mode. c, Hz distribution of GM TM22 mode. d, Hz distribution of GM TM32 mode. e, Hz distribution of GM TM41 mode. f Hz distribution of GM TM51 mode.

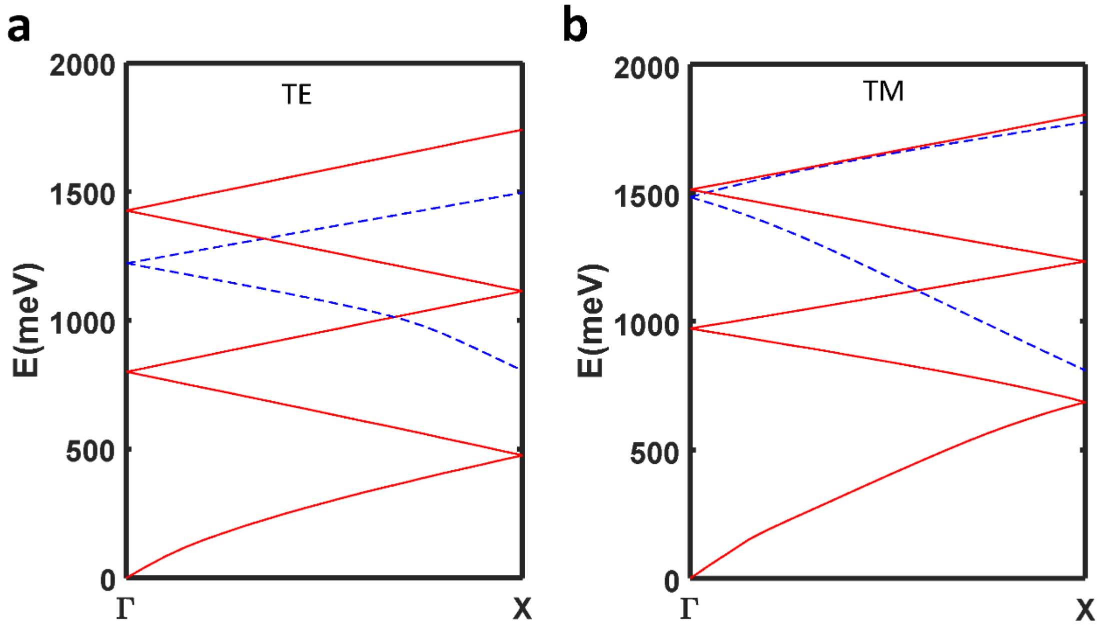

Figure S5. Band structure for a 1D perturbed meta-waveguide system. a, TE Band structure of a metagrating system. b, TM Band structure of a metagrating system. The thickness of top perturbation layer is chosen as 20 nm. The period is 548 nm and the width of the grating is 200 nm.

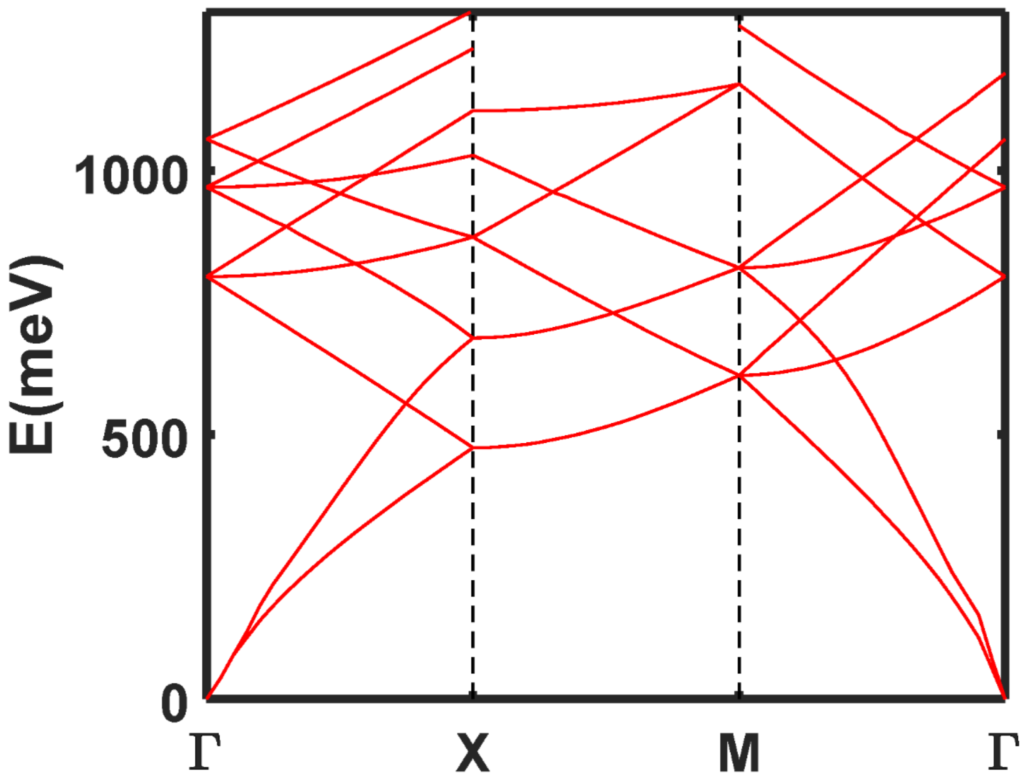

Figure S6. Band structure for a 3D perturbed meta-waveguide system. The periods along x and y axis are px = py = 548 nm. The top layer has a thickness 20 nm. The hole radius is 100 nm.

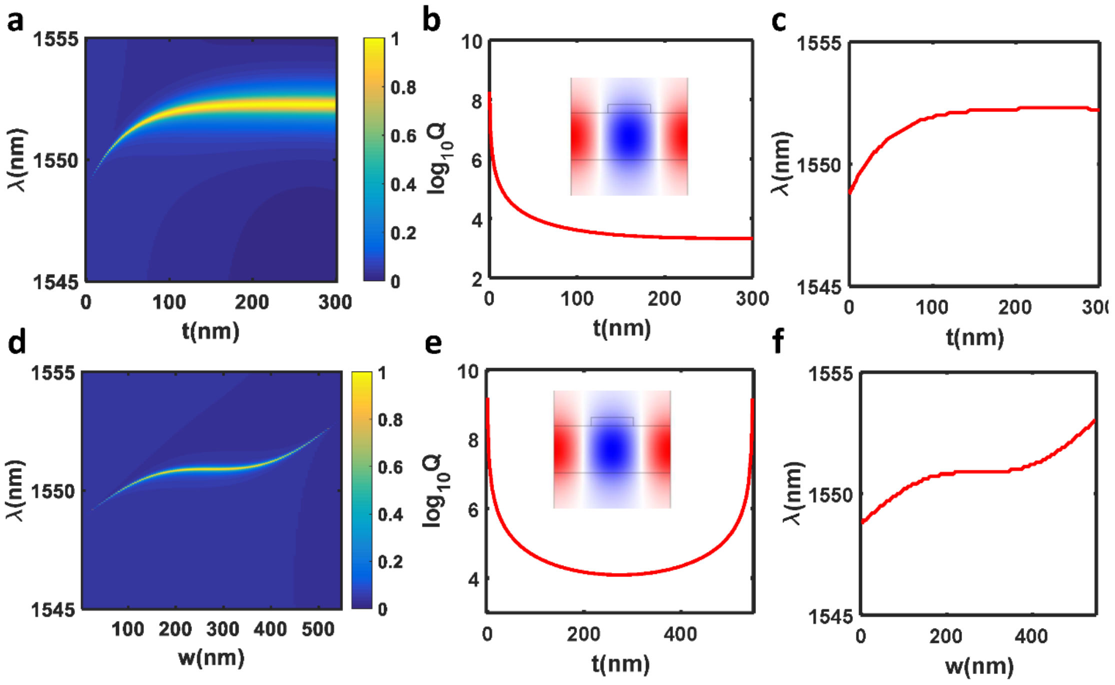

Figure S7. Properties of GMR TE31. a, Reflection mapping as functions of top layer thickness and incident wavelength for metagrating with p=548 nm and w=200 nm. b, Q-factor of GMR TE31 as a function of top perturbation layer thickness. c, Resonant wavelength of GMR TE31 vs top layer thickness. d, Reflection mapping as functions of grating width and incident wavelength for metagrating with p=548 nm and t=40 nm. e, Q-factor of GMR TE31 as a function of grating width. f, Resonant wavelength of GMR TE21 vs grating width.

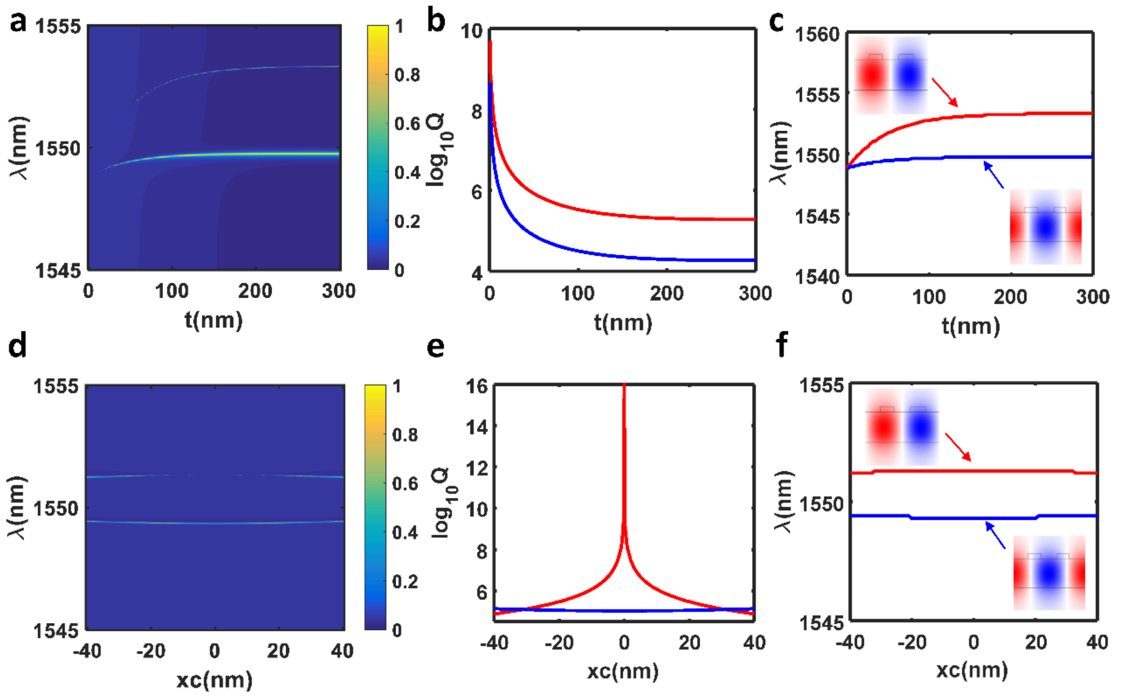

Figure S8. Properties of GMR TE21 and TE31 for metagrating with broken symmetry. a, Reflection mapping as functions of top layer thickness and incident wavelength. b, Q-factor of GMRs TE21 and TE31 as a function of perturbation layer thickness. c, Resonant wavelength of GMRs TE21 and TE31 vs top layer thickness. d, Reflection mapping as functions of gap center and incident wavelength. e, Q-factor of GMRs TE21 and TE31 as a function of gap center. f, Resonant wavelength of GMRs TE21 and TE31 vs gap center.

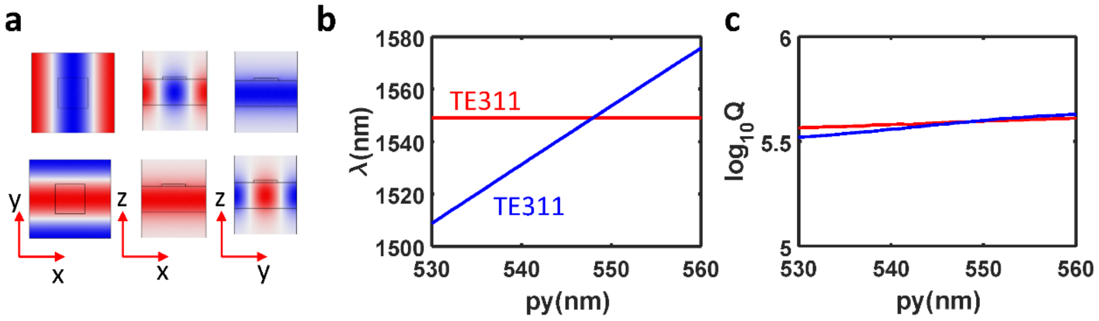

Figure S9. GMR in 3D metasurface. a, Electric field distribution for GMRs TE311 and TE131 mode. b, Resonant wavelength of modes TE311 and TE131 vs periods along the y-axis. c, Q-factors of modes TE311 and TE131 vs periods along the y-axis.

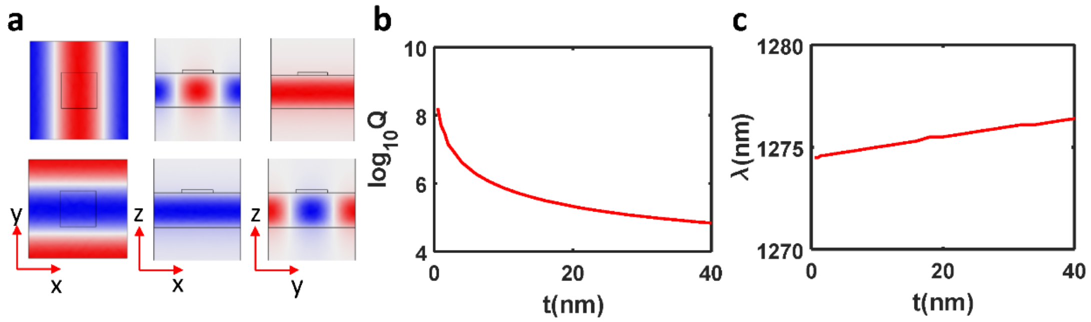

Figure S10. Properties of GMR in 3D metasurface. a, Electric field distribution of GMRs TE311 and TE131. b, Q-factors of GMRs TE311 and TE131 as a function of top layer thickness. c, Resonant wavelength of GMRs TE311 and TE131 as a function of top layer thickness

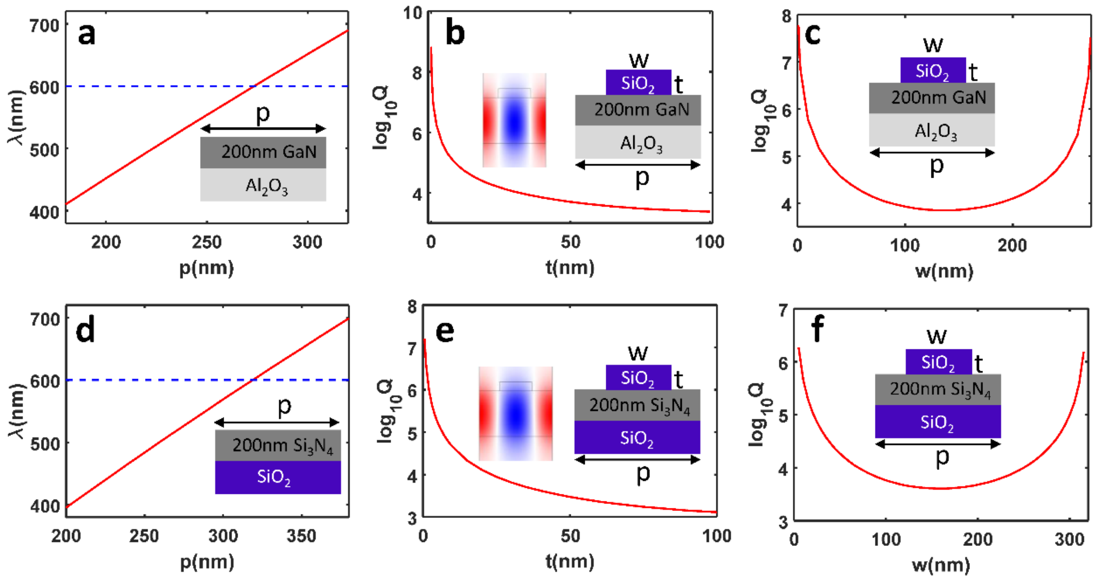

Fig.S11. High-Q GMR in the visible. a Guided mode wavelength versus the virtual period for 200 nm GaN on the sapphire substrate. The dashed line indicates the wavelength at 600nm. **b** Q-factor of mode TE31 versus the thickness of top SiO_2 grating width w=140 nm and p=273 nm. **c** Q-factor of mode TE31 versus the width of top SiO_2 grating with t=40 nm and p=273 nm. **d** Guided mode wavelength versus the virtual period for 200 nm Si_3N_4 on the glass substrate. The dashed line indicates the wavelength at 600 nm. **e** Q-factor of mode TE31 versus the thickness of top SiO_2 grating width w=140 nm and p=320 nm. **f** Q-factor of mode TE31 versus the width of top SiO_2 grating with t=40 nm and p=320 nm.

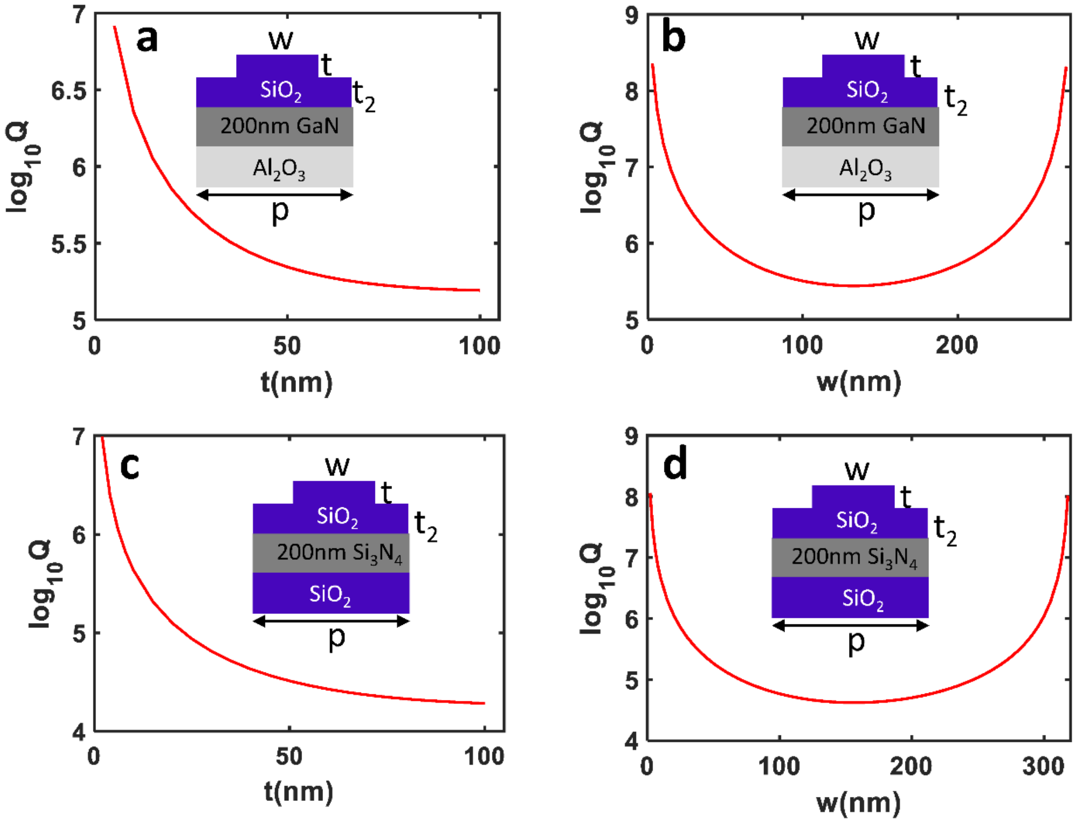

Fig.S12 High-Q GMRs in the visible. a Q-factor of mode TE31 versus the thickness of top SiO_2 grating with t_2=120 nm, w=140 nm and p=273 nm.The top grating sits on 200 nm GaN on the sapphire substrate. **b** Q-factor of mode TE31 versus the width of top SiO_2 grating with t=40 nm, t_2=120 nm and p=273 nm. **c** Q-factor of mode TE31 versus the thickness of top SiO_2 grating with t_2=120 nm, w=140 nm and p=320 nm. The top grating sits on 200 nm Si_3N_4 on the glass substrate. **d** Q-factor of mode TE31 versus the width of top SiO_2 grating with t=40 nm, t_2=120 nm and p=320 nm.

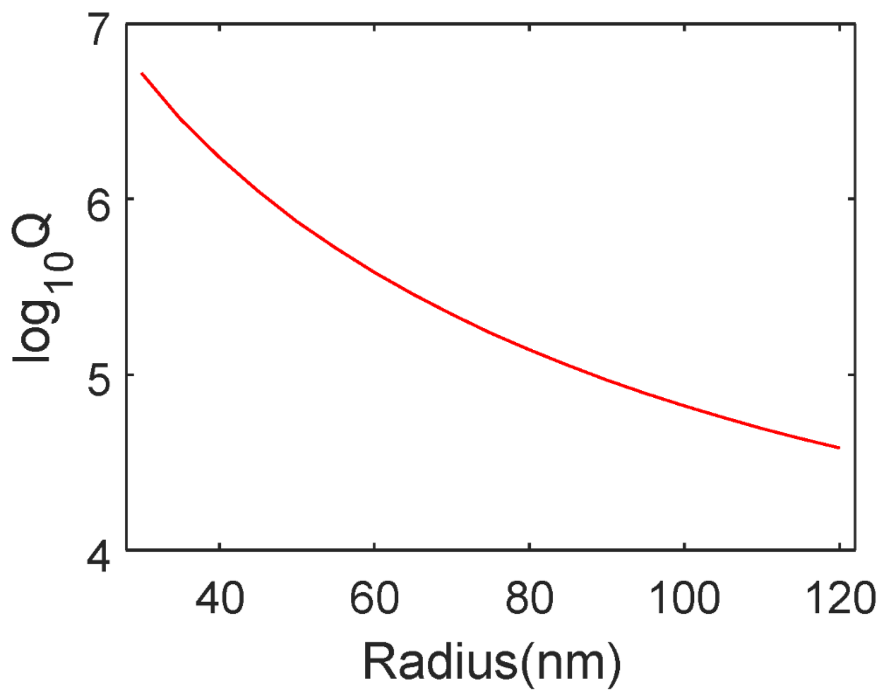

Figure S13. **Q-factor versus radius of resist photonic crystal slab on 220 nm SOI.**

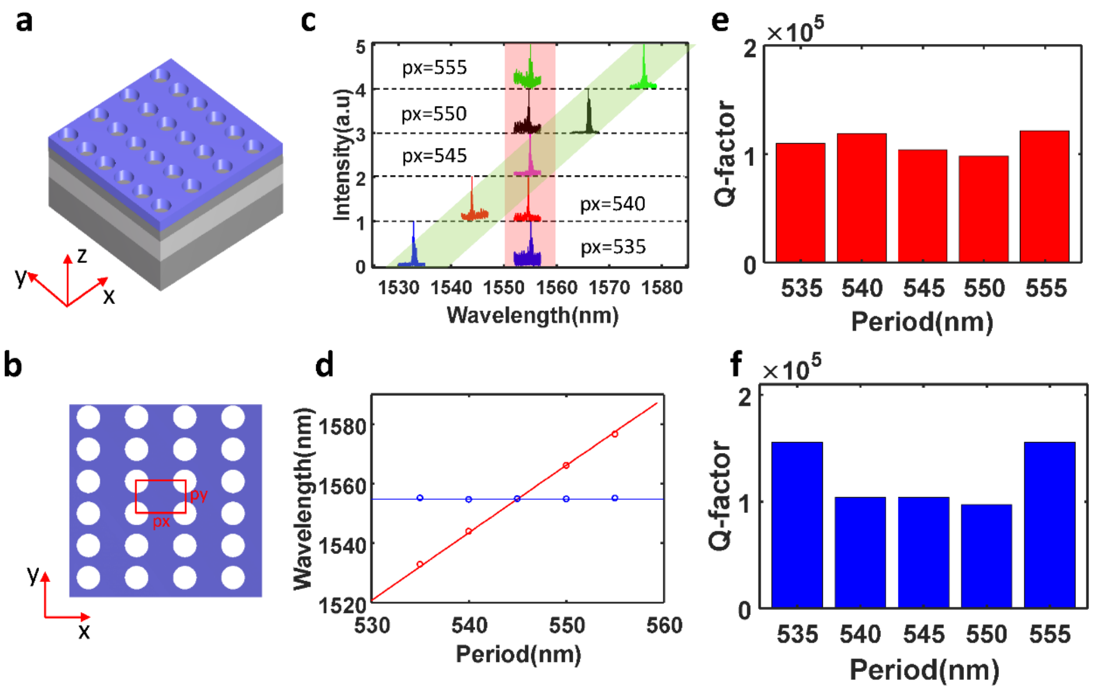

Figure S14. Experimental measurement of GMRs in 3D metasurface. a, Schematic drawing of the 3D metasurface. b, cross-section of metasurface. c, Measured reflection spectra of metasurfaces with different periods along the x-axis. The period along y axis is fixed as 545 nm. d, Retrieved resonant wavelength and calculated resonant wavelength for different periods. e, Measured Q-factor for mode TE_{311}, where the resonant wavelength is tuned by the varied px. f, Measured Q-factor for mode TE_{131} with stable resonant wavelengths.
